# Dbp5/DDX19 between Translational Readthrough and Nonsense Mediated Decay

**DOI:** 10.3390/ijms21031085

**Published:** 2020-02-06

**Authors:** Christian Beißel, Sebastian Grosse, Heike Krebber

**Affiliations:** Abteilung für Molekulare Genetik, Institut für Mikrobiologie und Genetik, Göttinger Zentrum für Molekulare Biowissenschaften (GZMB), Georg-August Universität Göttingen, 37077 Göttingen, Germany; christian.beissel@biologie.uni-goettingen.de (C.B.); sgrosse@gwdg.de (S.G.)

**Keywords:** translation, translation termination, mRNA quality control, NMD, Dbp5, Rat8, DDX19, mRNA degradation

## Abstract

The DEAD-box protein Dbp5 (human DDX19) remodels RNA-protein complexes. Dbp5 functions in ribonucleoprotein export and translation termination. Termination occurs, when the ribosome has reached a stop codon through the Dbp5 mediated delivery of the eukaryotic termination factor eRF1. eRF1 contacts eRF3 upon dissociation of Dbp5, resulting in polypeptide chain release and subsequent ribosomal subunit splitting. Mutations in *DBP5* lead to stop codon readthrough, because the eRF1 and eRF3 interaction is not controlled and occurs prematurely. This identifies Dbp5/DDX19 as a possible potent drug target for nonsense suppression therapy. Neurodegenerative diseases and cancer are caused in many cases by the loss of a gene product, because its mRNA contained a premature termination codon (PTC) and is thus eliminated through the nonsense mediated decay (NMD) pathway, which is described in the second half of this review. We discuss translation termination and NMD in the light of Dbp5/DDX19 and subsequently speculate on reducing Dbp5/DDX19 activity to allow readthrough of the PTC and production of a full-length protein to detract the RNA from NMD as a possible treatment for diseases.

Translational control is an organized and adaptable mechanism, which is vital for all organisms in life. The expression level and the quality of the protein expressed from a protein-coding gene depend both on the stability and on the quality of the expressed mRNA. Eukaryotic cells have evolved nuclear and cytoplasmic mRNA surveillance systems. Nuclear quality control captures transcripts that experience problems in 5′-capping, 3′ polyadenylation and/or intron splicing, prevents their nuclear export and initiates their degradation [1,2,3]. In contrast to that, the cytoplasmic quality control system ensures integrity of the mRNA open reading frame by monitoring ribosomal decoding. If this is not the case and the ribosome stalls without encountering a stop codon, those transcripts are eliminated by the no-go decay (NGD) and no-stop decay (NSD). When a premature termination codon (PTC) is detected, mRNA degradation occurs via the nonsense mediated decay (NMD) [4,5]. 

In this review we will summarize the process of translation termination with a focus on Dbp5/DDX19. We will then introduce the principles of NMD and explain the role of Dbp5/DDX19 between these two events. Finally, we will discuss why we think that Dbp5/DDX19 might be an interesting drug target to manipulate these processes and use this for potential treatments of diseases.

## 1. Translation Termination

On normal mRNAs, translation initiation is followed by elongation and ends with termination. Subsequently the ribosomes are split, which is termed ribosome recycling. These processes are linked through proteins that act comprehensively to maintain the homeostasis of the 40S and 60S ribosomal subunits, important for repeated rounds of translation. Initiation and elongation involve multiple players and several excellent reviews exist that describe the earlier phases of translation in detail [6,7,8]. Translation termination comprises three key events: (1) Recognition of the stop codon, (2) hydrolysis of the terminal peptidyl-tRNA bond and polypeptide chain release, and (3) ribosome recycling and disassembly of the termination complex. As soon as one of the three stop codons is reached on the RNA, the eukaryotic release factor eRF1 (encoded by *SUP45* in yeast) binds to the ribosomal A-site. eRF1 interacts with the GTPase eRF3 (encoded by *SUP35* in yeast), leading to polypeptide chain release and ribosome recycling [9]. eRF1 consists of three domains, which structurally mimic the shape of tRNAs with which it competes for binding to the ribosome. The N terminal domain, which comprises the YxCxxxF and TASNIKS motifs, is most important for recognition and binding to the stop codon [10,11,12]. The central domain on the other hand, especially the methylated GGQ motif, is necessary for the hydrolysis of the peptidyl-tRNA bond [13]. The C-terminal domain of eRF1 finally contacts eRF3, leading to its GTP-binding and subsequent hydrolysis and results in a conformational change important for proper termination [14,15]. Importantly, GTP hydrolysis of eRF3 leads to the dissociation of eRF1 and eRF3, allowing eRF1 to interact with Rli1 (ABCE1 in human) [6]. Rli1 not only functions in translation termination but also has an important function in ribosome splitting by forcing the ribosomal subunits apart through a tweezers-like movement upon NTP-hydrolysis. However, in termination the ordered binding of Rli1 to eRF1 after eRF3 release leads to conformational changes of eRF1, resulting in the aminoacyl bond hydrolysis [16,17,18,19]. 

During ribosome recycling, the ribosomes are split, resulting in free 60S subunit and a 40S subunit bound to mRNA and the deacylated tRNA, and subsequently, the post-termination complexes are disassembled. Recycling requires Rli1 to free the subunits and the eukaryotic initiation factors (eIF) 1, 1A and 3 to prevent reformation of the ribosome through occupying the post-recycled ribosomal subunits [20]. 

Most of the knowledge about termination and recycling was gained through in vitro assays and kinetic analyses in which nothing seemed to be missing. However, the situation in vivo must be different and regulation of these processes more complex because additional termination factors were discovered. Defects in termination, detected in termination readthrough assays, identified mutations in eIF5A and Pub1 that affect translation termination. Pub1 seems to fine tune termination in different nucleotide surroundings and eIF5A supports eRF1 activity in polypeptide chain release [21,22]. Research, mostly with *Sachharomyces cerevisiae*, identified additional important termination factors. Readthrough defects were furthermore detected in mutants of the initiation factors eIF3 and Hcr1 [23,24] and the DEAD-box RNA helicase Dbp5 [25]. The initiation factors eIF3 and Hcr1 function in the release of the termination complex, which is why Hcr1 is not only considered as bona fide initiation factor anymore. It was shown, that Hcr1, which is delivered by eIF3, releases eRF3-GDP from the ribosome after termination [16,23,24]. The function of Dbp5 in translation termination was surprising. Not only because it has an additional well-known function in mRNA export but also because identification of its function abrogated the prior view that eRF1 and eRF3 would enter the ribosome together.

## 2. The Function of Dbp5 in Translation Termination

**Figure 1:**
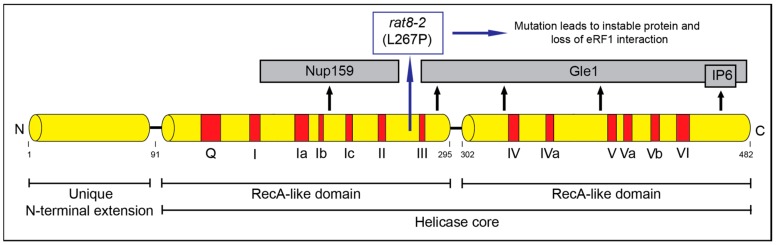
Scheme of the domain structure of Dbp5. Indicated are the domains important for RNA- and ATP binding (red) and the domains important for the interaction partners and co-factors (grey).

The DEAD-box protein Dbp5 has a well-established function in mRNA export from the nucleus to the cytoplasm [25,26,27,28,29]. Dbp5 (encoded by *RAT8* in yeast and DDX19 in humans) is conserved and essential in all eukaryotes. It acts as an RNA helicase with an ATP dependent RNA- and protein complex remodeling activity [27,30]. Dbp5 belongs to the helicase superfamily 2 (SF2) and contains 13 characteristic sequence motifs and the eponymous sequence Asp-Glu-Ala-Asp (DEAD) in motif 2 (Figure 1) [31]. Dbp5 is localized in the nucleus, in the cytoplasm and concentrated around the nuclear rim [27,32]. A nuclear export signal and a nuclear import signal were identified in the N-terminus of the protein, enabling shuttling between the compartments [33]. The helicase core of Dbp5 is composed of two highly conserved RecA-like domains linked by a hinge region [34]. The unique N-terminal extension of Dbp5 is important for its autoregulation and determines the specificity of the enzyme [35].

In mRNA export, Dbp5 displaces the mRNA export receptor heterodimer Mex67-Mtr2 from the appearing transcript on the cytoplasmic side of the nuclear pore complex (NPC) [36]. Located at the cytoplasmic filaments of the NPC, Dbp5 interacts with the N-terminal domain of its ADP release factor NUP159/Rat7 for recycling, enabling multiple rounds of Mex67-dissociation [27,37,38]. Under physiological conditions, Nup159 is not the only co-factor required for Dbp5 activity but also Gle1-IP_6_ [38,39]. Gle1-IP_6_ preferentially binds to ATP-Dbp5 and causes a conformational change to initiate ATPase activity [40,41]. ATP bound Dbp5 shows the highest affinity for single stranded RNA, and the presence of a non-hydrolysable ATP analog leads to tightly bound RNA in vitro [38,42]. Thus, by binding to Gle1, the ATPase- and remodeling-activity of Dbp5 is triggered and the subsequent conformational change allows Nup159 binding and recycling of the helicase [29,40,43,44]. Besides mRNA export, Dbp5 was identified to participate in the nuclear export of pre-ribosomal subunits and tRNA; however, its role in these processes is less well understood [33,45].

The function of Dbp5 in translation termination was first identified in 2007 [25]. Dbp5 was detected in polysomes and mutants of *DBP5* were shown to be hypersensitive to translational inhibitors. In addition, physical interaction of Dbp5 and eRF1 was shown [25]. Subsequently, also Gle1 was identified to co-localize with translating ribosomes and identified as an interaction partner of eRF1 [40,46]. Most importantly, mutations in *DBP5* and *GLE1* caused severe stop codon readthrough defects [25,40]. Likewise, it was recently shown that the human protein DDX19 stabilizes translation termination complexes and participates in termination [47]. 

**Figure 2:**
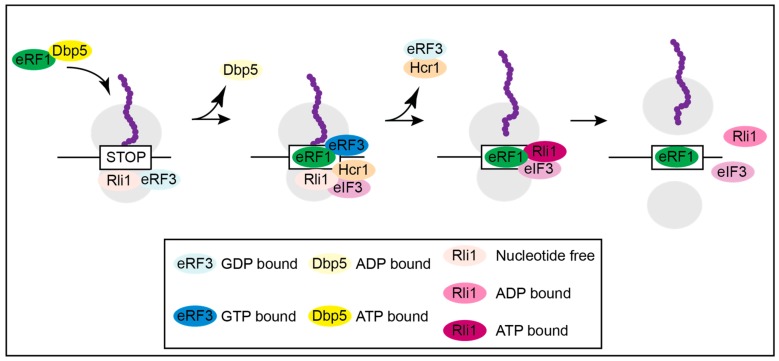
Model for translation termination. The yeast DEAD-box RNA helicase Dbp5 delivers eRF1 to the ribosome located on a stop codon. Its function is to prevent an early contact of eRF1 with eRF3. As soon as eRF1 was placed properly, the ATP-hydrolysis of Dbp5 dissociates the helicase. Free eRF1 subsequently contacts eRF3 that waited in its GDP-bound form attached to Rli1. This contact triggers GTP-binding of eRF3 and subsequent hydrolysis causing polypeptide chain and tRNA release. At the same time, eIF3 delivers Hcr1, which eliminates eRF3 from the complex. Removal of eRF3 enables contact of eRF1 with Rli1-ATP and through ATP-hydrolysis the ribosomal subunits are split.

Subsequent in vivo and in vitro studies suggest that Dbp5 regulates a stepwise assembly of the termination complex (Figure 2) and [16]. Although not all details are clear and established, the following model suggests the most likely mechanism for translation termination. Unlike what was anticipated in older models, eRF1 and eRF3 presumably do not enter termination together as a complex, but rather Rli1 and eRF3-GDP associate with the ribosome first as soon as the A-site is unoccupied. Subsequently, nucleotide free Rli1 promotes the binding of the Dbp5-eRF1 complex, which formed before in the cytoplasm. After delivery of eRF1 through the helicase Dbp5 and its proper positioning in the A-site of the ribosome, which requires ATP hydrolysis, Dbp5-ADP dissociates. For its recycling Dbp5 moves to the NPC, where it releases the ADP and re-binds to ATP through the actions of Nup159 and Gle1-IP6. Dbp5-ATP then captures a new eRF1 molecule, preparing for the next round of translation termination [16]. The double duty of Dbp5 in mRNA export and in termination, not only couples these important processes but also shows how efficient nature uses one factor for several functions. Remarkably, Gle1 seems to stay longer at the ribosome than Dbp5 and presumably has an additional function in ribosome recycling or translation initiation. This is independent of Dbp5 and IP_6_, as Gle1 interacts with the eIF3, which was not seen for Dbp5 [16,43,46].

The scenario at the ribosome after Dbp5 has delivered eRF1 and has left the termination complex continues with the interaction of eRF1 and eRF3 (Figure 2). Their interaction is prevented as long as Dbp5 is bound, because both proteins bind to the C-terminus of eRF1, and their binding is mutually exclusive. In fact, competition experiments revealed that eRF3 is not able to dissociate a preformed Dbp5-eRF1 complex [16]. In this way, the interaction of eRF1 and eRF3 is prevented until eRF1 was properly positioned. This is most important to prevent a premature contact, because as soon as these proteins interact with each other, eRF3 has a higher affinity to GTP, which instantly triggers GTP hydrolysis and dissociation of eRF1 and eRF3. eRF3-GDP is removed from the termination complex by Hcr1 [16,23,24]. Such a regulatory principle, the shielded delivery of eRF1 by Dbp5, makes sense, when considering the situation in mutants of *DBP5*, in which eRF1 does not bind and is therefore not delivered to the ribosome by the helicase. 

## 3. Mutations in *DBP5* Lead to Termination Readthrough

**Figure 3:**
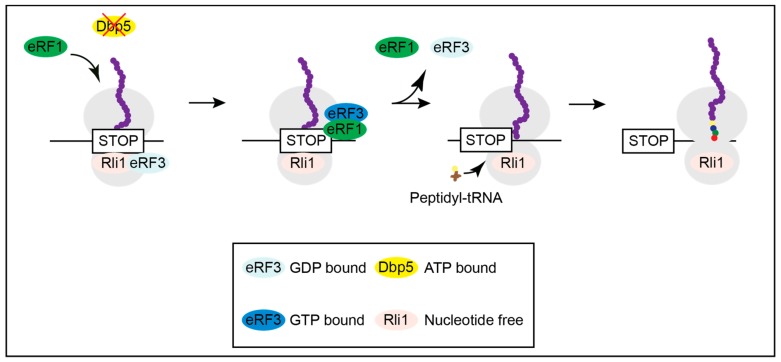
Lack of Dbp5 leads to translational readthrough. Mutations in Dbp5 prevent a shielded entry of eRF1 to the ribosome, resulting in an early contact of eRF1 and eRF3 and their subsequent dissociation. This increases the probability for the incorporation of a near-cognate tRNA and the continuation in translation.

When Dbp5 is not functional, as shown in several mutants, eRF1 approaches the ribosome on its own and contacts eRF3 before it is properly positioned for termination, resulting in the immediate dissociation of both release factors and translational readthrough [16]. Inefficient termination increases the chance of the incorporation of a near-cognate tRNA and results in ongoing translation and longer proteins (Figure 3) and [16].

In most cases, general readthrough might be problematic for cells, however, tuning readthrough also holds the possibility for regulation. Such a potential regulation is still unclear and not much is known for a possible role of Dbp5. However, for example during stress, Dbp5 localizes to the nuclei of cells [48] and is thus depleted from the cytoplasm. Its absence presumably causes translational readthrough at stop codons, allowing the synthesis of longer polypeptides. This could create proteins with new functions, such as proteins that in their longer form now contain a nuclear localization signal or a degradation signal, which can change their place of function or eliminate this protein function altogether. In multicellular organisms, Dbp5/DDX19 might even be developmentally regulated, e.g., by phosphorylation or other modifications, for such purposes under normal conditions. These aspects certainly need more research for clarification.

## 4. Regulatory Principles in Nonsense Mediated Decay (NMD)

For more than 25 years, one major goal has been to understand the mechanisms underlying NMD. Although at first only considered as a cytoplasmic mRNA quality control system, current research has clearly identified its power in regulating the RNA-transcriptome beyond RNA surveillance, e.g., in biological contexts such as development [49,50]. Therefore, it is of particular interest to identify the parameters that in the cells discriminate mRNAs that should be translated from those that should be degraded. Clearly, NMD is linked to inefficient translation termination [51]. However, when and why this can occur is not fully understood. Not only the RNA sequence itself is relevant but also the proteins within the ribonucleoparticle (RNP).

**Figure 4:**
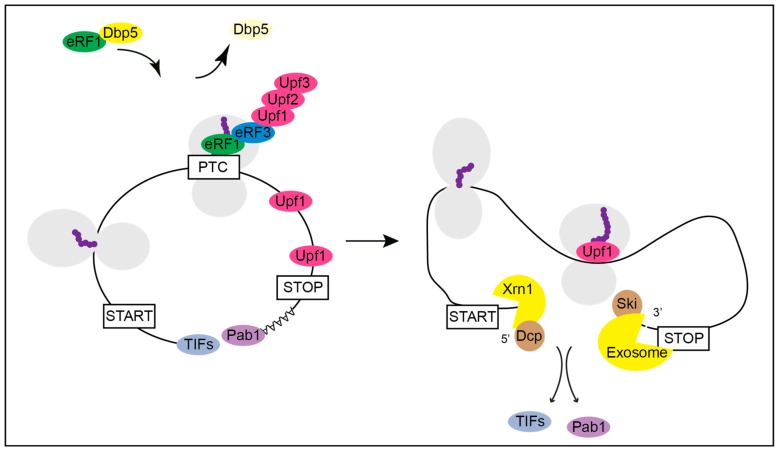
Termination at a premature termination codon (PTC) and the subsequent initiation of the nonsense mediated decay (NMD) of the mRNA. Under normal conditions yeast Dbp5 promotes the stop codon recognition by eRF1 and eRF3 also at a PTC. However, when recognized as premature by the NMD machinery, Upf1 binds to the release factors and assembles the Upf1-2,3 complex. Consequently, further translation is inhibited, and downstream factors are recruited to promote rapid mRNA decay. The decapping factors Dcp1 and Dcp2 and Xrn1 act from 5′ to 3′ and the Ski complex and the exosome degrade the PTC-containing mRNA from 3′ into 5′ direction.

Generally, in mRNA surveillance, a premature termination codon (PTC) is distinguished from a normal one as the trigger for NMD. NMD can only be initiated during translation termination as it requires the decoding of a stop codon by eRF1 and eRF3 [52]. At a PTC, the ATPase and helicase Upf1 interacts with eRF1 and eRF3 to initiate the NMD pathway (Figure 4) and [53,54]. Upf1 is activated by its less abundant co-factors Upf2 and Upf3 [55,56]. These trigger a conformational change in Upf1 to increase its ATPase and Helicase activity, which is initially inhibited by eRF1 and eRF3 [53,57]. In higher eukaryotes SMG1 (and its co-factors SMG8 and SMG9) binds to UPF1 to form the SURF (SMG1 UPF1 Release Factors) complex [58,59]. Then, the helicase DHX34 promotes the association with UPF2 and UPF3 to form the DECID (decay inducing) complex [60]. Further, SMG1 phosphorylates UPF1 [61], which is considered to be the step that commits an mRNA to NMD [62]. In *S. cerevisiae* phosphorylation of Upf1 was also described, but there the relevance is not clear [63,64]. After formation of the Upf1-2,3 complex (or DECID complex in higher eukaryotes), Upf1 triggers rapid mRNA degradation.

Of course, the NMD pathway should only be initiated when translation termination occurs prematurely. Several models have been proposed to describe the differentiation of a normal stop codon and a PTC. The two main models are the exon junction complex (EJC) induced NMD and the long 3′UTR model [65]. The former is based on the position of the EJC, a complex that is placed during splicing near the exon–exon junction [66]. Stop codons typically occur in the last exon and have no downstream EJC in the 3′UTR. If, however, translation is terminated at a stop codon that is followed by an EJC, the complex strongly promotes NMD [67,68,69]. It serves as a binding platform for UPF2 and UPF3 and, if downstream of a termination event, promotes their interaction with the SURF complex [58,70,71] The long 3′UTR model, which is more relevant in yeast but also described in higher eukaryotes, focuses on the distance between a stop codon and the poly(A) tail. A normal stop codon is typically in proximity to the poly(A) tail and the poly(A) binding protein Pab1 (PABP in human). Pab1 interacts with eRF3, promotes regular termination and prevents NMD [51,72,73]. At a PTC the distance to Pab1 is increased, which allows the formation of the Upf1-2,3 complex and the initiation of NMD. Further, Upf1 ubiquitously binds any mRNA and is displaced by the translating ribosome. As a consequence, Upf1 stays bound to the 3′UTR. It was suggested that longer 3′UTRs have more bound Upf1 molecules, which might promote the Upf1 binding to the terminating ribosome [74,75]. In addition to these two models, it was found that the sequence downstream (and in a few cases upstream) of the stop codon further promotes or inhibits NMD [76,77,78,79].

Although there is growing insight into the mechanisms by which NMD is initiated and degrades transcripts, there are still many open questions. It is for instance unknown how PTC-detection is signaled to the ends of the transcript, where degradation is initiated. In this context, the mRNP conformation during NMD is presumably important but not yet understood. For the EJC induced NMD in humans, it was described that Upf1 interacts with the cap binding complex (CBC) in a process where the mRNA might fold back so that the Upf1 complex at the PTC can reach the 5′ end [80,81]. In yeast NMD, however, where the 3′ UTR length is the main determinant, the CBC is dispensable [82]. Moreover, NMD was shown to occur also on transcripts in which CBC has dissociated and instead the cap binding protein and translation initiation factor eIF4E has bound [76,83]. Presumably several proteins are involved in the required mRNP remodeling for NMD, but this is to date almost completely unknown.

In the EJC independent initiation of NMD, it is unclear how Upf2 and Upf3 join Upf1. In yeast, it is assumed that they must be specifically recruited to NMD targets, due to their low cellular abundance. However, their association with translated mRNPs is Upf1 independent [84], suggesting an involvement of other currently unknown NMD factors. The sequences that affect NMD, and proteins that bind to these sequences are poorly understood, but these appear to affect both the EJC induced NMD and long 3′ UTR induced NMD in yeast as well as in metazoans [76,77,78,79].

NMD of different targets presumably requires a different subset of proteins; even the co-factor Upf2 is not required for all NMD [65,67]. Which proteins are functioning in which instances of NMD and how this is regulated are still very hazy matters. Future research will have to identify new NMD factors and sort out their functions on different NMD targets. In particular research in simple organisms such as baker’s yeast can help to untangle the underlying basic principles of NMD.

## 5. Termination Readthrough and Nonsense Mediated Decay (NMD)

Another aspect is important in this regard. Many inherited human diseases and cancer types are caused by the loss of a specific protein due to the elimination of its transcripts that contained a premature termination codon (PTC) [85,86,87]. In fact, one-third of inherited human diseases are due to PTCs, and such premature stops in translation account for up to 70% of all genetic disorders [88,89,90]. Such PTC containing transcripts can result from, e.g., splicing defects or mutations in the DNA. Usually, such PTC containing mRNAs are recognized and eliminated by nonsense mediated decay, so that the protein is never made. However, some PTC-containing transcripts escape NMD and produce truncated, dominant, negatively operating gene products that can also cause diseases [85,91].

It was observed that overexpressing Upf1 alleviates the negative effects in a TDP-43 induced rat model of the neurodegenerative disease ALS [92]. Thus, promoting NMD in such cases might be a valuable therapeutic approach. However, in some cases, the remaining functional allele is haploinsufficient, and NMD cannot regain the functional protein of a PTC containing transcript [93]. In these cases, readthrough of the PTC may recover the loss of function. When a PTC is read through, a near cognate tRNA is inserted at the stop codon, and translation continues until it is terminated by the correct termination codon [94]. Hence, the produced protein has, if at all, only one amino acid exchange and can, at least partially, recover the loss of function. Indeed, for several targets it was reported that nonsense suppressors increase the amount of functional protein [95]. Such an approach is also potentially interesting for cancer research as some types of cancer carry nonsense mutations in tumor suppressors [96,97,98], which may regain function through readthrough. Generally, nonsense suppression as a therapeutic approach is encouraged by the fact that PTCs are more susceptible to read through than normal stop codons, presumably due to reduced termination efficiency at a PTC [51,95]. Research with suppression agents revealed that global translation is unaffected at concentrations that cause read through at PTCs [95,99,100]. This does not preclude, however, the possibility of side effects of nonsense suppression therapy as NMD is also involved in regulatory mechanisms that include wild typical PTC-containing mRNAs [101].

Attempts were started to downregulate NMD, allowing the production of some of the full-length protein as treatment for diseases. Such drugs could act as nonsense suppressors (e.g., Ataluren or RTC13). These compounds can be natural or synthetic aminoglycosides and nonaminoglycosides [100,102,103,104,105,106]. Interestingly, several compounds have already been identified to inhibit the helicase and ATPase activities of the Plasmodium falciparum Dbp5/DDX19 homolog PfD66 [107]. Although promising, a great concern with these compounds is that they lack drug target specificity. Moreover, the many gaps and weaknesses in current NMD models urge for further research of the mechanisms and participating factors in NMD. 

**Figure 5:**
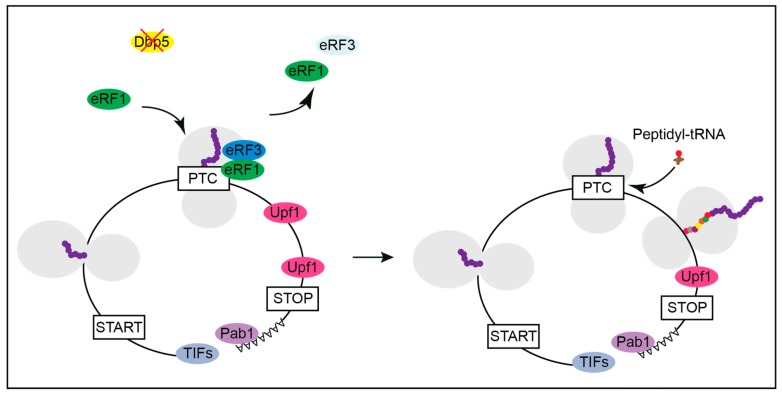
Affecting the availability of functional Dbp5 might generate full length proteins from PTC containing mRNAs. With a reduced function of Dbp5, the stop codon recognition of eRF1 and eRF3 is impaired. If the PTC is not recognized as a stop codon, a near cognate peptidyl-tRNA enters the ribosome, and translation elongation continues. A normal stop codon is less susceptible to translational readthrough than a PTC, presumably due to additional factors that promote translation termination, such as Pab1. Hence, there is a high probability that translation can terminate at the correct stop codon rather than at the PTC. This will most likely generate a full-length protein with only one amino acid exchange instead of a truncated polypeptide.

Further, different NMD targets do not respond similarly to one nonsense suppressor. Ataluren (also called PTC124), a therapeutic for Duchenne muscular dystrophy, was not approved for Cystic Fibrosis, as clinical trials did not show sufficient improvement [108,109]. This highlights the necessity to investigate further and for different approaches in nonsense suppression. Here Dbp5/DDX19 might serve as an interesting new drug target to fine tune translation read through, possibly in conjunction with other drugs in lower doses (Figure 5).

Strategies to repress NMD for correct protein production are one hope for therapy. Understanding the full mechanistic details of translation termination and NMD in the cellular context will be important and necessary to develop more sophisticated therapies for PTC-caused diseases.
